# HIV-Infected Children Have Lower Frequencies of CD8+ Mucosal-Associated Invariant T (MAIT) Cells that Correlate with Innate, Th17 and Th22 Cell Subsets

**DOI:** 10.1371/journal.pone.0161786

**Published:** 2016-08-25

**Authors:** Alka Khaitan, Max Kilberg, Adam Kravietz, Tiina Ilmet, Cihan Tastan, Mussa Mwamzuka, Fatma Marshed, Mengling Liu, Aabid Ahmed, William Borkowsky, Derya Unutmaz

**Affiliations:** 1 New York University School of Medicine, Department of Pediatrics, Division of Infectious Diseases and Immunology, New York, NY, United States of America; 2 New York University, Department of Microbiology, New York, NY, United States of America; 3 Bomu Hospital, Mombasa, Kenya; 4 New York University, Division of Biostatistics, Department of Population Health, Department of Environmental Medicine, New York, NY, United States of America; 5 The Jackson Laboratory for Genomic Medicine, Farmington, CT, United States of America; Karolinska Institutet Department of Medicine Solna, SWEDEN

## Abstract

Mucosal-associated invariant T cells (MAIT) are innate T cells restricted by major histocompatibility related molecule 1 (MR1) presenting riboflavin metabolite ligands derived from microbes. Specificity to riboflavin metabolites confers MAIT cells a broad array of host-protective activity against gram-negative and -positive bacteria, mycobacteria, and fungal pathogens. MAIT cells are present at low levels in the peripheral blood of neonates and gradually expand to relatively abundant levels during childhood. Despite no anti-viral activity, MAIT cells are depleted early and irreversibly in HIV infected adults. Such loss or impaired expansion of MAIT cells in HIV-positive children may render them more susceptible to common childhood illnesses and opportunistic infections. In this study we evaluated the frequency of MAIT cells in perinatally HIV-infected children, their response to antiretroviral treatment and their associations with HIV clinical status and related innate and adaptive immune cell subsets with potent antibacterial effector functions. We found HIV+ children between ages 3 to 18 years have significantly decreased CD8+ MAIT cell frequencies compared to uninfected healthy children. Remarkably, CD8 MAIT levels gradually increased with antiretroviral therapy, with greater recovery when treatment is initiated at a young age. Moreover, diminished CD8+ MAIT cell frequencies are associated with low CD4:CD8 ratios and elevated sCD14, suggesting a link with HIV disease progression. Last, CD8+ MAIT cell levels tightly correlate with other antibacterial and mucosa-protective immune subsets, namely, neutrophils, innate-like T cells, and Th17 and Th22 cells. Together these findings suggest that low frequencies of MAIT cells in HIV positive children are part of a concerted disruption to the innate and adaptive immune compartments specialized in sensing and responding to pathogenic or commensal bacteria.

## Introduction

Mucosal-associated invariant T cells (MAIT) are a recently described unconventional T cell subset that plays an important role in antibacterial and antifungal innate immune responses in the peripheral blood and at mucosal surfaces [1–3]. MAIT cells express a semi-invariant TCRα chain, Vα7.2, with a narrow TCRβ repertoire [4–6]. These innate T cells are restricted by major histocompatibility complex related molecule, MR1 [7]. MR1 is an antigen-presenting molecule found ubiquitously in numerous cells and tissues, but selectively expressed at the cell surface [8]. When presented with microbe-derived riboflavin (vitamin B2) metabolite ligands bound to MR1 molecules, MAIT cells become activated and mount cytotoxic and inflammatory immune responses [9]. MAIT cells have been evolutionarily conserved across species, with over 80% sequence homology between mammalian MR1 genes, suggesting a crucial role in immunity [8, 10, 11]. Phenotypic markers for MAIT cells include Vα7.2 TCR expressed with CD161. A majority of MAIT cells are CD8+ or CD4-CD8- T cells, while a small percentage are CD4+ T cells [4, 12, 13]. In the peripheral blood, MAIT cell frequency varies, ranging from 0.5–10% of T lymphocytes and up to 25% of CD8+ T cells in healthy adults [12, 14, 15]. As their name suggests, MAIT cells predominantly localize to mucosal surfaces including the gut lamina propria, lung, and liver [3, 7, 12].

Specificity for riboflavin metabolite ligands allows MAIT cell responses to encompass diverse gram-positive and -negative bacteria, mycobacteria, and yeast [1–3, 16, 17]. The crucial role of MAIT cells during infection was demonstrated in MAIT cell knockout mice, which, after infection with *Klebsiella pneumonia*, developed higher bacterial loads and increased mortality [18]. Further studies in these mice found delayed clearance of *Mycobacterium abscessus* and *Escherichia coli* [1]. *In vitro*, MAIT cells react to microbes that metabolize riboflavin, including Pseudomonas, Klebsiella, Staphylococcus and Candida [1, 17]. *Ex vivo* studies of humans with *Mycobacterium tuberculosis* infection demonstrate MAIT cells detect cells infected with *M*. *tuberculosis* as well as *E*. *coli*, *Salmonella typhimurium*, *and S*. *aureus* [3]. Upon stimulation, MAIT cells have the capacity to kill infected cells via secretion of cytolytic enzymes, perforin and granzyme, and to produce inflammatory cytokines, IFNγ, IL-17, TNFα, and IL-2, which stimulate dendritic cell maturation and recruitment of conventional CD4+ and CD8+ T cells [9, 12, 19–21]. Thus, MAIT cells simultaneously exert effector innate functions while soliciting adaptive immune responses. During bacterial infections, MAIT cells diminish in the peripheral blood and localize to tissues [1, 3]. At these sites, it remains unclear how MAIT cells discriminate between pathogens and commensal organisms, which both may have the capacity to stimulate them via riboflavin metabolites. Beyond responses to infection, multiple studies link loss of MAIT cells to inflammatory conditions, including autoimmune disorders such as inflammatory bowel disease and systemic lupus erythematosus, type II diabetes mellitus, and obesity, suggesting a regulatory role for this subset [22–28].

While MAIT cells react to phylogenetically diverse bacteria and fungi, they do not mount responses to viral infections. Nonetheless, during HIV infection, MAIT cells are rapidly depleted from both the peripheral blood and gut mucosa [15, 29–36]. The peripheral loss of MAIT cells is irreversible, whereas the gut mucosal MAIT cell population appears to be restored after antiretroviral treatment [15, 29, 31, 32]. Similarly, in SIV infected rhesus macaques, MAIT cells are systemically depleted in peripheral blood, mesenteric lymph nodes, and lung mucosa [37]. With their potent anti-microbial activity, this decrease of MAIT cells during HIV infection may leave individuals particularly vulnerable to opportunistic infections such as *M*. *tuberculosis*. Additionally, depletion of MAIT cells in the gut may promote microbial translocation due to a compromised mucosal barrier and contribute to systemic immune activation. Indeed, colonic MAIT frequencies in HIV+ adults correlated with systemic immune activation [31].

MAIT cell development begins in the fetal thymus with positive selection. CD161++ naïve cells are present in cord blood then differentiate into memory cells in the first few months of infant life [12, 13, 38]. In mice mature memory MAIT cell expansion in the periphery requires B cells and commensal microbial flora [13]. However, in human fetal tissue, activated MAIT cells are present in mucosal sites before commensal microbiota develop [39]. In healthy adults, B cells activate MAIT cells by presenting riboflavin metabolites of either commensal or pathogenic bacteria [40]. While MAIT cell expansion occurs during infancy and early childhood, few studies have evaluated MAIT cells within pediatric cohorts in health or disease [14, 24, 27, 41, 42]. In perinatally-infected HIV+ children, clear disruptions in the immune system begin in infancy with depletion of CD4+ T cells and systemic immune activation. We assessed MAIT cell frequencies in perinatally HIV-infected children, their response to antiretroviral therapy, and their associations with HIV clinical markers. In addition we evaluated correlations between MAIT cell levels and innate-like T cell subsets, and other immune cells with potential antibacterial effector functions, such as Th17 cells, in perinatally HIV-infected children. We found peripheral CD8 MAIT cell frequencies in HIV+ children were decreased compared to HIV negative children, and gradually increased with antiretroviral therapy, particularly when started at a young age. Diminished CD8 MAIT levels were present in HIV+ children with low CD4:CD8 ratios and elevated sCD14, suggesting a link with HIV disease progression. Furthermore, CD8 MAIT cells correlated with antibacterial innate and adaptive immune subsets, namely neutrophils, γδT, NKT, Th17 and Th22 cells.

## Materials and Methods

### Participants

Ethical approval for this study was obtained from New York University (10–02586) and Kenyatta National Hospital / University of Nairobi (P283/07/2011). Written informed consent, and verbal assent when appropriate, was obtained from all participants and/or parents. We enrolled a total of 98 perinatally-infected HIV+ and 52 HIV negative-unexposed children ages 3–18 years old from Bomu Hospital in Mombasa, Kenya between 2011–2012. HIV+ children included 49 antiretroviral therapy naïve (ART-) and 49 HIV+ children on antiretroviral treatment for at least six months (ART+). Individuals with a recent acute illness, active *Mycobaterium tuberculosis* or malaria infection, or pregnancy within one year were not eligible for study entry.

Plasma and peripheral blood mononuclear cells (PBMC) were isolated from peripheral blood by centrifugation and Ficoll-Hypaque (GE Healthcare) density gradient centrifugation and then cryopreserved in -80°C and liquid nitrogen respectively.

HIV-, ART-, and ART+ were matched for age and gender (Table 1). Median percent and absolute CD4 in HIV- children were 37% and 974 cells/mm^3^ respectively. ART- had median CD4% of 24%, absolute CD4 cell count of 583 cells/mm^3^, and HIV viral load of 4.9 log copies/ml. ART+ had median CD4%, absolute CD4 cell count, and HIV viral load of 32%, 1014 cells/mm^3^, and 2 log copies/ml respectively (Table 1; complete subject characteristics in S1 Table).

**Table 1:** Subject Characteristics.

	HIV-	HIV+ ART-	HIV+ ART+	p
**n**	52	49	49	
Age*	11 (6–13)	9 (7–13)	10 (4–12)	NS^a^
**Female**	23	24	24	NS^b^
%CD4*	37 (30–40)	24 (13–29)	32 (26–37)	p<0.0001^a^
**CD4 cells/mm**^**3**^	974 (734–1305)	583 (367–880)	1014 (718–1387)	p = 0.0003^a^
log HIV copies/ml*		4.9 (4.3–5.3)	2 (2–2.5)	p<0.0001^c^

*Median values with upper and lower quartile range

^a^ Kruskal-Wallis test

^b^ Chi-Square test

^c^ two-sided Mann-Whitney test

#### Laboratory testing

A complete blood cell count was performed on whole blood at Bomu Hospital in the clinical laboratory to obtain percent neutrophil and lymphocyte values. HIV RNA PCR was performed on diluted plasma samples with Roche, COBAS^®^ AmpliPrep/COBAS^®^ TaqMan^®^HIV-1 Test, version 2.0. Limit of detection was 110 copies/ml.

### Flow cytometric studies

Cryopreserved PBMCs were thawed then evaluated with 10-color flow cytometry. Cells were stained with fixable viability dye (eBioscence) in PBS for 30 minutes, washed, then stained with fluorescent-conjugated antibodies at 4°C for 30 minutes in PBS buffer containing 2% FCS and 0.1% sodium azide. For intracellular cytokine staining, cells were activated with Phorbol ester (20ng/mL; Sigma), Ionomicin (500ng/mL; Sigma), and monensin (GolgiStop; BD Biosciences) for 5 hours, then fixed, permeabilized (eBioscience kit per manufacturer's instructions) and stained with intracellular antibodies. Stained cells were analyzed using LSRII flow cytometer (BD Bioscience) and FlowJo software (Tree Star). The following anti-human antibodies were used (fluorochrome; clone): CD3 (Alexa 700; UCHT1), CD4 (BV510; OKT4), CD8 (PE Cy7; RPA-T8), CD161 (BV 421; HP-3G10), Vα7.2 (PE; 3C10), γδTCR (BD: biotin; B1; secondary streptavidin BV 605), Vα24Jα18 (APC; 6B11), CD45RO (APC Cy7;), IFNγ (eBioscience: PE Cy7; 4S.B3), IL-17A (BV 421; BL168), IL-22 (eBioscience: efluor 710; 22URTI). All antibodies were from Biolegend except when noted. To identify HIV specific CD8 T cells, PBMCs of HLA-A*0201+ subjects were stained with MHC dextramer SLYNVATL (Immudex) and CD3, CD8, and Vα7.2 antibodies according to manufacturer's instructions. Singlet lymphocytes were gated based on forward and side scatter properties. Dead cells were excluded based on viability dye. All populations are reported as percent of CD8+, CD4-CD8-, or CD4+ T cell parent populations.

### Plasma sCD14

Plasma levels of sCD14 were quantified by ELISA assay using Human CD14 Duoset kit (R&D Systems) per manufacturer’s instructions. Plasma was diluted 1500 fold and each test performed in duplicate. Results reported are the average of duplicate results.

### Statistical analysis

All statistical analyses were performed using GraphPad Prism software. Continuous variables were summarized using median and inter quartile range (IQR) and categorical variables were summarized using counts and proportions. Comparisons between participant categories were computed with the two-sided Mann-Whitney test. Data from multiple time points were evaluated with the paired Wilcoxon matched-pairs signed rank test. Correlations were assessed with the Spearman’s rank test. For linear regression analysis, fold change in CD8 MAIT frequencies (post-/pre-ART MAIT or T1/T0 MAIT) and log-transformed CD8 MAIT percentages were treated as dependent variables, and independent variables included age at ART initiation and total years on ART. Threshold of significance for all tests was 0.05.

## Results

### Decreased frequency of circulating CD8 MAIT cells in HIV-infected children

**Fig 1:**
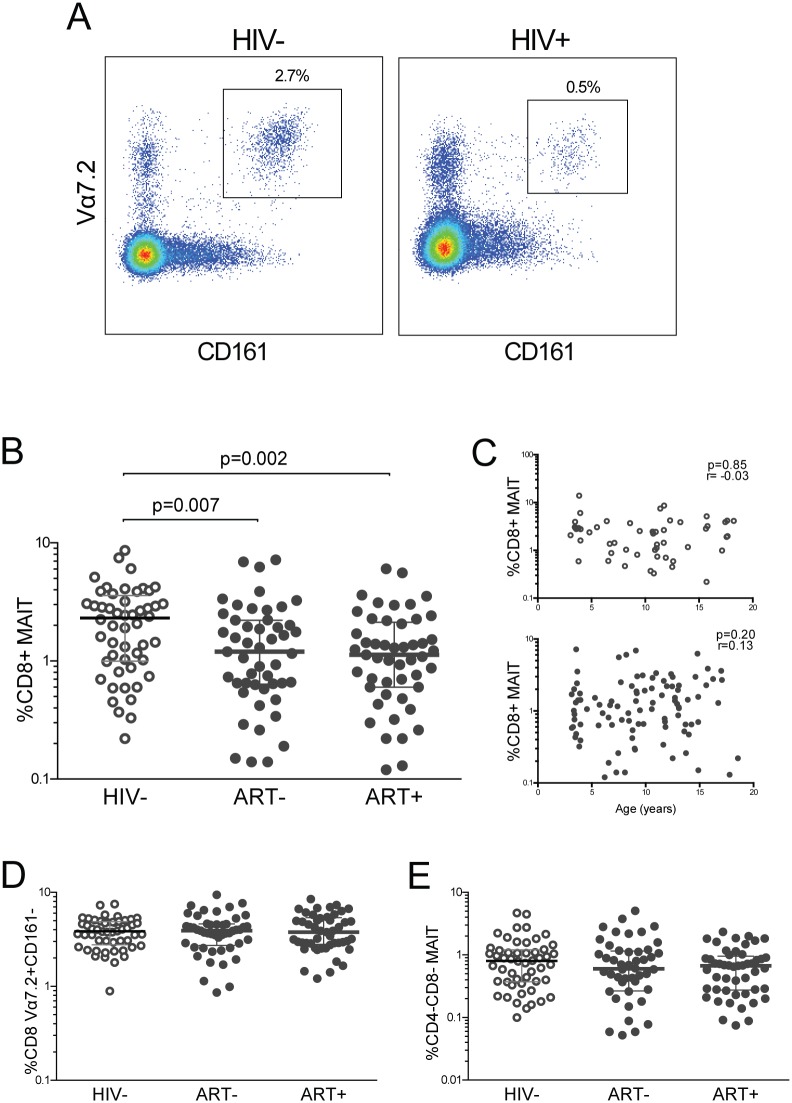
MAIT cells in HIV infected and uninfected children. (A) FACS plot showing representative gating to identify CD8 MAIT cells in an HIV- and HIV+ subject. Plot shown is gated on CD8+ T cells. MAIT cells are identified as the Vα7.2^+^CD161^+^ population. (B) CD8+ MAIT cells in HIV-, ART-, and ART+ children. (C) CD8+ MAIT cells vs. age in years in HIV- (open circles) and HIV+ (filled circles) children. CD8+ Vα7.2+CD161- non-MAIT cells (D) and CD4-CD8- Vα7.2^+^CD161^+^ MAIT cells (E) in HIV-, ART-, and ART+ children.

We identified MAIT cells within CD8+ T cells by gating on CD161^+^Vα7.2^+^ cells (MAIT cells) in HIV- and HIV+ children (Fig 1A). Untreated HIV+ children had a decrease in MAIT cells (median 1.2%, IQR 0.64–2.2%) compared to HIV- children (median 2.3%, IQR 1–3.6%). ART+ children also had markedly reduced MAIT cells with a median of 1.1% of CD8+ T cells (IQR 0.6–2.1%, Fig 1B). These MAIT cells were not HIV specific CD8 T cells, as they were negative for HIV peptide SLY by dextramer staining (S1 Fig). In addition, there was no significant correlation between the frequency of CD8 MAIT cells and age in either HIV- or HIV+ children (Fig 1C). As an internal control, CD8+ T cells expressing Vα7.2 and CD161 negative (non-MAIT) cells were similar in HIV- and HIV+ children (Fig 1D) [36]. Next we evaluated the proportion of CD161^+^Vα7.2^+^ cells in the CD4-CD8- subset, and found no significant difference between frequencies in HIV- and HIV+ children (Fig 1E). HIV- and HIV+ subjects had similar levels of total MAIT cells when expressed as a percent of T cells (S2A Fig) or lymphocytes (S2B Fig) and as absolute numbers (S2C Fig). Hence there was a perturbation of MAIT cells specifically within the CD8+ T cell compartment in HIV+ children.

### CD8 MAIT cells gradually increase with ART in HIV+ children

**Fig 2:**
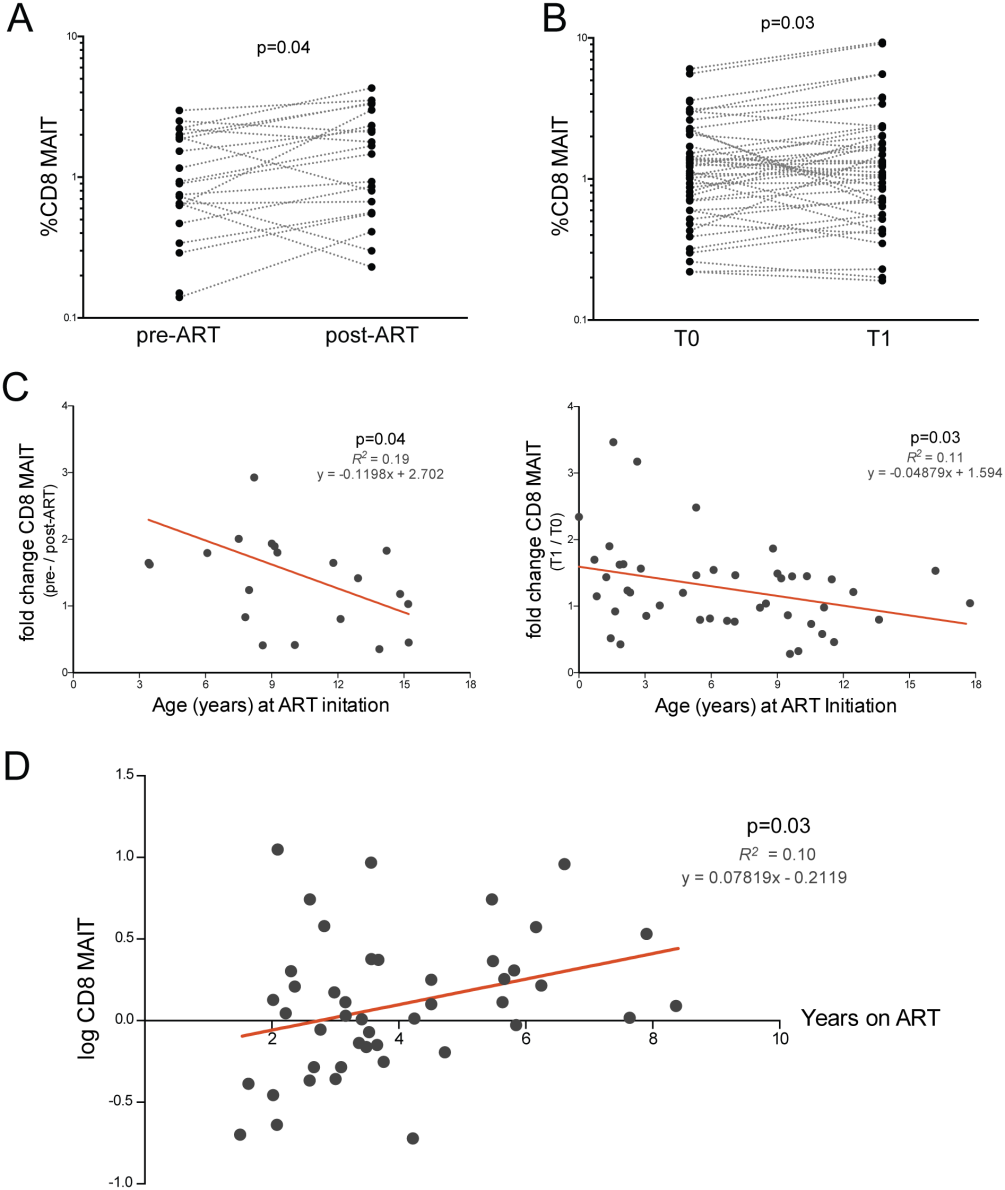
CD8 MAIT cells gradually recover with ART in HIV+ children. (A) CD8+ MAIT cells in ART- children before (pre-ART) and 10–21 months after ART initiation (post-ART). (B) CD8+ MAIT cells in ART+ children at baseline (T0) and at 10–21 month follow-up visit (T1). (C) Age in years at the time of ART initiation vs. fold change in %CD8 MAIT cells in ART- (post- / pre-ART CD8 MAIT; left graph) and ART+ children (T1/T0 CD8 MAIT; right graph). (D) Log CD8+ MAIT cells in ART+ children vs. total duration of ART in years.

Previous studies of HIV+ adults found that depleted MAIT cells in the peripheral blood are not restored after the initiation of ART [15, 29]. Therefore, we evaluated whether HIV+ children who began ART were able to recover their MAIT population. We assessed MAIT frequencies before (pre-ART) and 10–21 months after antiretroviral treatment initiation (post-ART) in the ART- cohort. These subjects showed a response to ART with an increase in percent of CD4+ T cells and a decrease in HIV viral load (p<0.0001; S3 Fig). Remarkably there was a small but significantly higher CD8 MAIT cell frequency after ART, with a median difference of 0.27 between pre- and post-ART CD8 MAIT levels (p = 0.04, Fig 2A). We also prospectively examined MAIT cells in ART+ children at their initial visit (T0), then at a 10–21 month follow-up visit (T1), and again found a slight increase (median difference 0.15, p = 0.03, Fig 2B). Next we evaluated whether initiation of ART at a younger age improved restoration of MAIT cells with a linear regression model using age at ART initiation to predict fold change in CD8 MAIT cells after ~1 year on antiretroviral therapy. For both ART- children beginning treatment, and ART+ children continuing therapy, earlier ART initiation was associated with a larger increase in CD8 MAIT frequencies (ART-: p = 0.04, *R*^*2*^ = 0.19; ART+: p = 0.03, *R*^*2*^ = 0.11; Fig 2C). Lastly, we assessed whether frequencies of MAIT cells increased with prolonged antiretroviral therapy. In ART+ children, longer duration of ART predicted higher CD8 MAIT cell levels (p = 0.03, *R*^*2*^ = 0.1, Fig 2D). Thus CD8 MAIT cell frequencies slowly increase with antiretroviral therapy in HIV+ children, with greater expansion when ART is started at a younger age.

### CD8 MAIT cells and markers of HIV disease progression in HIV-infected children

**Fig 3:**
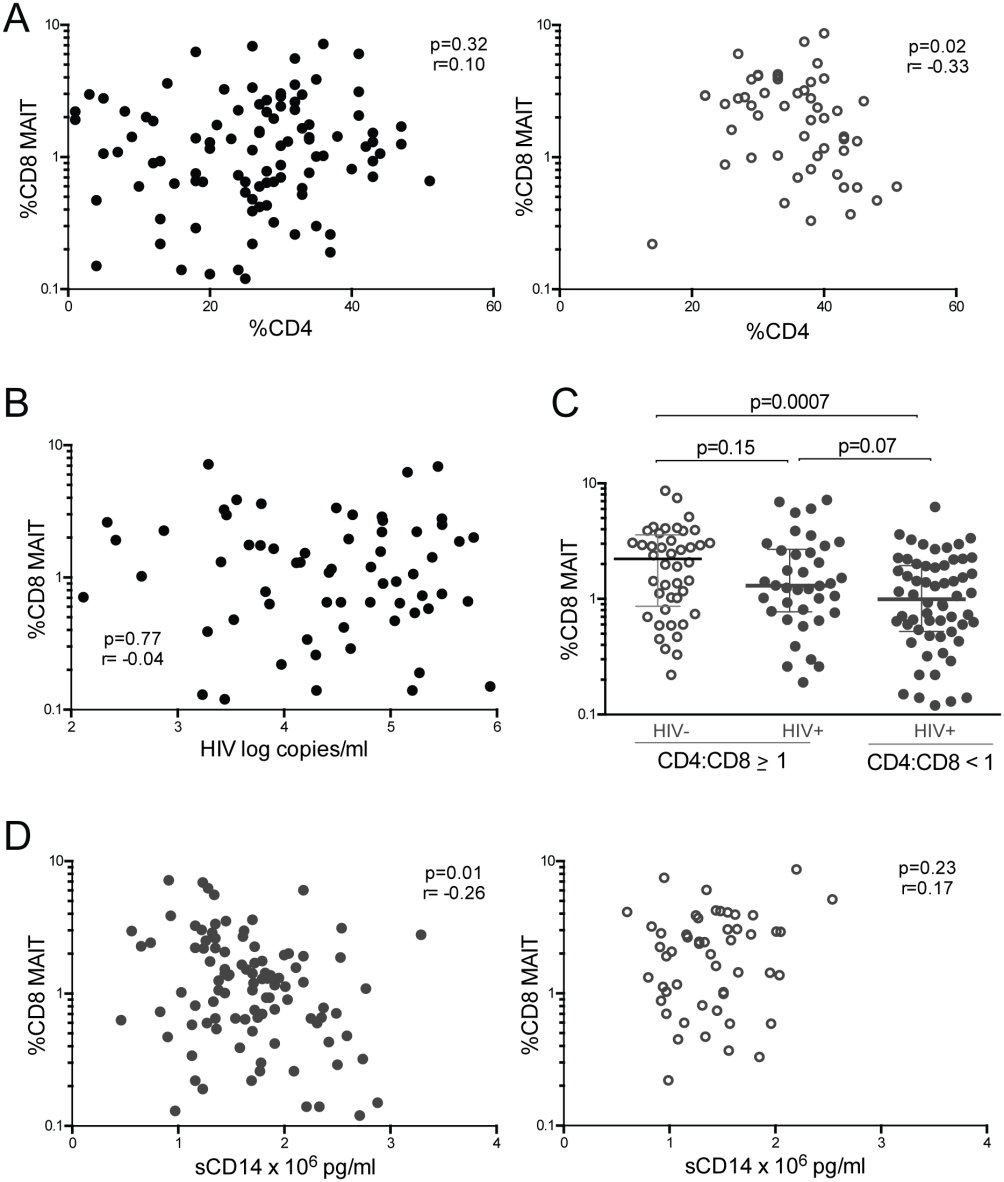
CD8 MAIT cells and HIV disease progression. (A) Correlation between CD8+ MAIT cells and %CD4+ T cells in HIV+ (filled circles) and HIV- (open circles) children. (B) Percent CD8+ MAIT cells vs. HIV log copies/mL in viremic HIV+ children. (C) Comparison between CD8+ MAIT cells in HIV- and HIV+ children divided into groups with CD4:CD8 ratios greater than or equal to one and ratios less than one. (D) CD8+ MAIT cells vs. plasma sCD14 levels in HIV+ (closed circles) and HIV- (open circles) children.

To determine the relation between MAIT cells and HIV clinical measures, we evaluated whether MAIT cells correlated with the percent of CD4 T cells in HIV- and HIV+ children (Fig 3A). HIV negative children exhibited an inverse correlation between CD8 MAIT cells and percent of CD4 T cells, while in HIV+ children this correlation was not present (Fig 3A). In viremic HIV+ children, we found no association between HIV viral load and CD8+ MAIT cells (Fig 3B). During HIV infection, in addition to CD4 T cell depletion, CD8 T cells rise, and the ratio of CD4:CD8 T cells, which is normally above one, is inverted to less than one. This CD4:CD8 ratio has been shown to predict disease progression and mortality in HIV+ adults [43]. Interestingly, CD8 MAIT cells are lower specifically in HIV+ children with CD4:CD8 ratios less than one compared to HIV- children (Fig 3C). Another predictor of HIV disease progression is plasma sCD14 level, which results from monocyte activation after stimulation by lipopolysaccharide [44]. In HIV+ children, CD8 MAIT cells inversely correlated with plasma sCD14 levels (Fig 3D). This correlation was absent in HIV- children (Fig 3D). Thus the reduction of CD8 MAIT cells in HIV+ children directly linked with predictors of HIV mortality, low CD4:CD8 ratios and elevated plasma sCD14.

### CD8 MAIT cells correlate with innate and innate-like immune cells in HIV+ children

**Fig 4:**
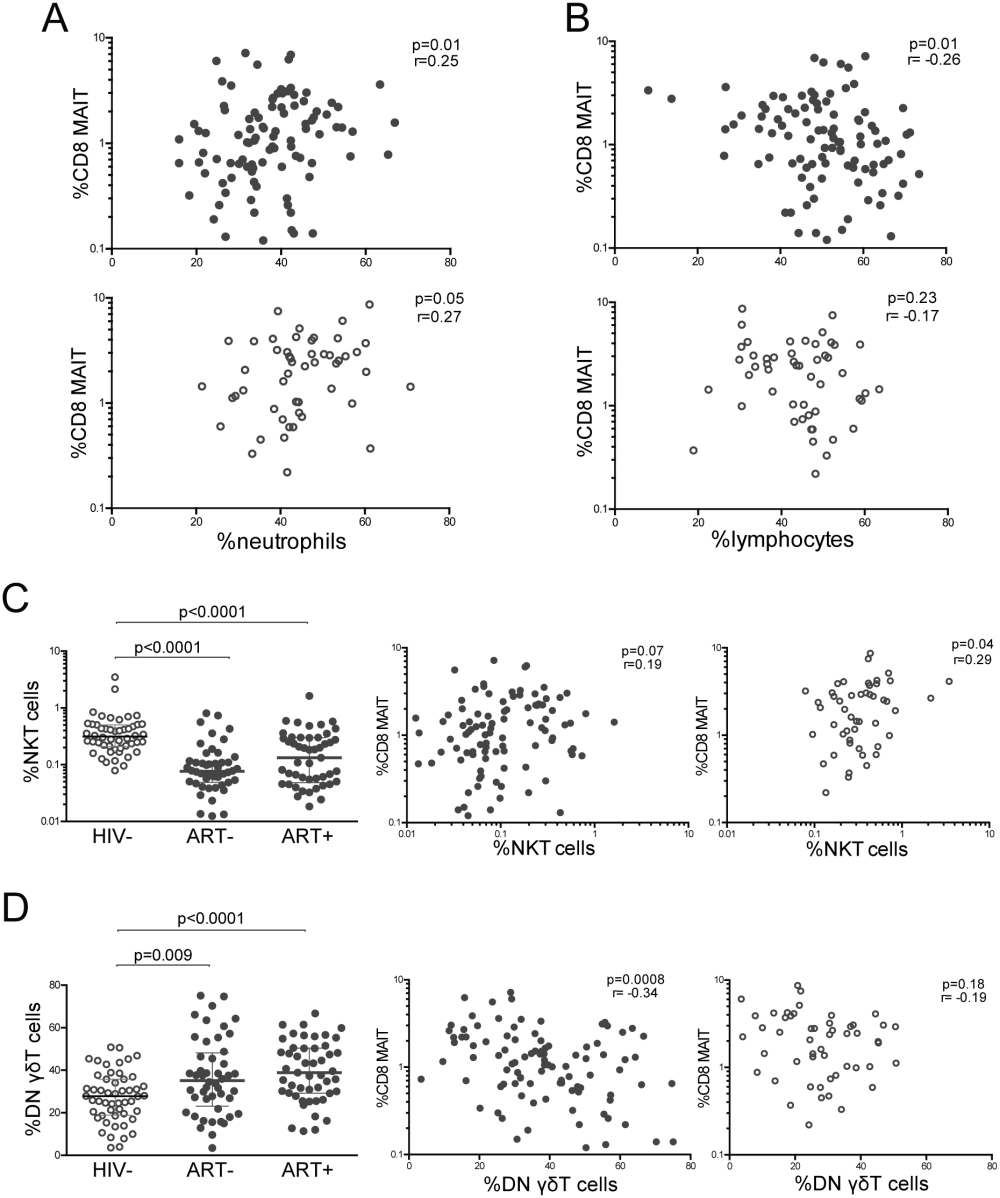
MAIT cells correlate with innate and innate-like immune cells in HIV-infected children. Correlation graphs showing percent of CD8+ MAIT cells vs. percent of (A) neutrophils and (B) lymphocytes in HIV+ (closed circles) and HIV- (open circles) children. (C) A comparison of NKT cells as a percent of total T cells in HIV-, ART-, and ART+ children is shown in the left graph. NKT cells were identified by gating on Vα24Jα18^+^ cells within CD3+ T cells. On the right are correlation graphs between CD8+ MAIT and NKT cells in HIV+ (closed circles) and HIV- (open circles) children. (D) γδT cells were identified by gating on γδTCR^+^ cells within CD4-CD8- (DN) T cells. Graphs show a comparison of DN γδ T cell in HIV-, ART-, and ART+ children, and their correlation with CD8+ MAIT cells in HIV+ (closed circles) and HIV- (open circles) children.

We next sought to evaluate whether MAIT cells relate to other innate or innate-like cells that mediate host defense against bacterial pathogens. First, we determined that in HIV+ children, the proportion of CD8 MAIT cells positively correlated with the percent of circulating neutrophils, which are the primary first responders to bacterial infections (Fig 4A). Conversely, CD8 MAIT cells had a negative correlation with peripheral lymphocytes in HIV+ children (Fig 4B). These correlations between CD8 MAIT cells and neutrophils or lymphocytes were not present in HIV- children (Fig 4A and 4B). Next we evaluated invariant natural killer T cells (NKT), which are also innate-like T cells that recognize non-peptide glycolipid antigens presented by CD1d molecule [45]. We identified NKT cells by expression of Vα24Jα18 TCR on T cells (S4A Fig). Similar to CD8 MAIT cells, both treated and untreated HIV+ subjects had markedly lower NKT cells compared to HIV- children (Fig 4C). Accordingly, there was a positive correlation between CD8 MAIT cells and NKT cells in HIV- children with a similar trend in HIV+ children (Fig 4C). Finally, we assessed CD4-CD8- (DN) γδ T cells, which also mount innate-like responses, exhibit cytolytic activity, and secrete inflammatory cytokines in response to bacterial pathogens (gating strategy in S4B Fig) [45]. In contrast to MAIT cells, the frequency of DN γδ T cells was higher in HIV+ children compared to HIV- children, and inversely correlated with MAIT cells (Fig 4D). CD8 MAIT cells did not correlate with DN γδ T cells in HIV- children (Fig 4D). Thus, reduced levels of MAIT cells in HIV-infected children, showed strong associations with other innate and innate-like cell subsets that also mediate sensing and responses to bacterial infections.

### Reduced CD8 MAIT cell frequencies coincide with loss of Th17 and Th22 cell subsets in HIV-infected children

**Fig 5:**
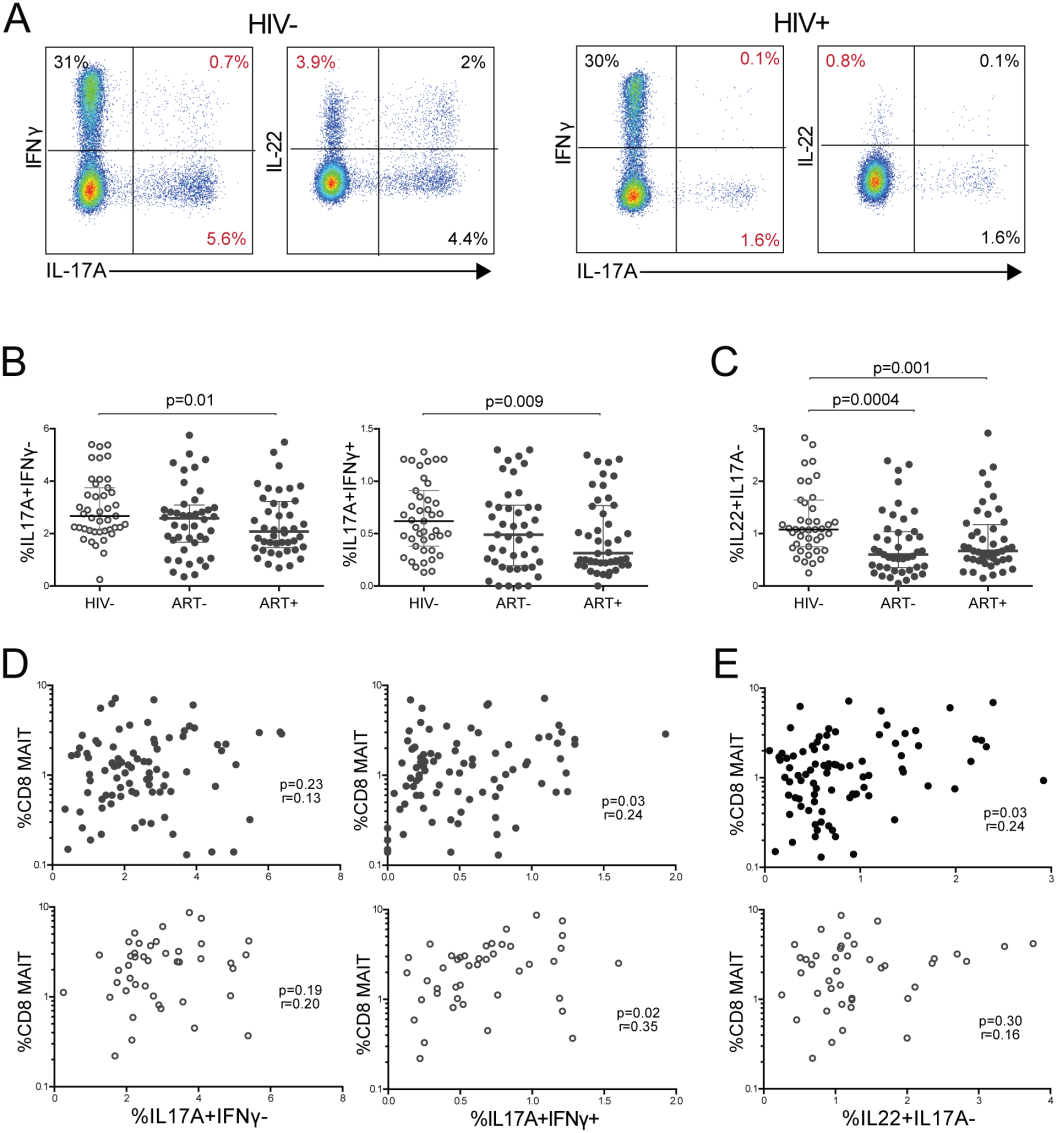
Lower CD8 MAIT cell frequencies correlate with loss of Th17 and Th22 subsets in HIV+ children. (A) FACS plot showing representative gating to identify Th17 and Th22 subsets in an HIV- and HIV+ subject. Plots shown are gated within CR45RO+ (memory) CD4+ T cells. (B) Comparisons of IL-17A^+^IFNγ^-^ and IL-17A^+^IFNγ^+^ memory CD4 T cells in HIV-, ART-, and ART+ children. (C) Comparisons of IL-22^+^IL-17A^-^ memory CD4 T cells in HIV-, ART-, and ART+ children. Correlations between CD8+ MAIT cells and (D) IL-17A^+^IFNγ^-^ and IL-17A^+^IFNγ^+^ memory CD4 T cells and (E) IL-22^+^IL-17A^-^ memory CD4 T cells in HIV+ (closed circles) and HIV- (open circles) children. All cytokine populations were gated within memory CD4+ T cells.

MAIT cells predominantly exert their functions at mucosal sites [12]. Likewise, Th17 and Th22 cells release tissue-protective cytokines, have a preference for mucosal surfaces, and exert antibacterial effector functions [46–48]. As such we compared Th17 and Th22 cells in HIV- and HIV+ children and evaluated for correlations with MAIT cells. We first identified Th17 subsets as IFNγ^-^IL-17A^+^ and IFNγ^+^IL-17A^+^ subsets and the Th22 subset as IL-17^-^IL-22^+^ cells within memory (CD45RO^+^) CD4+ T cells (Fig 5A). ART+ children had lower levels of IL-17A producing T cells both negative and positive for IFNγ production (Fig 5B). Th22 cells (IL-17^-^IL-22^+^) were lower in treated and untreated HIV+ children compared to HIV- children (Fig 5C). We next evaluated whether these mucosal T cell subsets, Th17, Th22, and MAIT cells, were associated. Remarkably, MAIT cells correlated with double-positive (IFNγ^+^IL-17A^+^) but not single-positive (IFNγ^-^IL-17A^+^) Th17 cells in HIV+ children (Fig 5D). The correlation between MAIT cells and IL-17^+^IFNγ^+^ Th17 cells was also present in HIV- children (Fig 5D). ART+ children also had decreased Th1 (IFNγ^+^IL-17^-^) cells compared to HIV- children, but there was no significant association between MAIT and Th1 cells in either HIV- or HIV+ children (S5 Fig). Finally, Th22 cells correlated with MAIT cells in HIV+ children, but not HIV- children (Fig 5E). Together, these findings suggest MAIT cells may be disrupted in concert with mucosal-protective antibacterial CD4+ T cell subsets during HIV-infection.

## Discussion

In this study we demonstrated perinatally HIV-infected children between ages 3 to 18 years have significantly lower frequencies of peripheral CD8 MAIT cells regardless of antiretroviral treatment. Remarkably, these CD8 MAIT cells gradually increase with antiretroviral therapy, with greater restoration when ART is initiated at a young age. Additionally, lower MAIT cell frequencies occur in HIV+ children with inverted CD4:CD8 ratios and associate with rising sCD14, suggesting a link between MAIT cell depletion and HIV disease progression. Finally, the frequency of CD8 MAIT cells tightly correlates with neutrophils, innate-like T cells, and mucosa-protective Th17 and Th22 subsets, which are endowed with potent antibacterial effector functions.

To our knowledge this is the first report of MAIT cells in HIV-infected children and the first description of MAIT frequencies in a healthy cohort of HIV negative children from sub-Saharan Africa. Among our healthy control pediatric subjects the median MAIT frequency was 2.3% of CD8 T cells and 0.9% of CD3+ T lymphocytes, whereas medians reported in children of similar ages in the United States and Ireland were ~6% of CD8 T cells ~2% of CD3+ T lymphocytes [24, 27]. These lower median values in our cohort may reflect higher burden of endemic bacterial and parasitic infections, ethnicity, or genetic factors. Indeed, previous studies of HIV+ adults from South Africa and Malaysia also reported lower median MAIT frequencies in healthy controls compared to that reported by Leeansyah and Cosgrove in western nations [15, 29, 30, 34]. Perhaps endemic bacterial infections trigger migration of MAIT cells to affected tissues, leading to lower circulating levels even in HIV negative healthy control subjects. In our cohort, both HIV negative and positive subjects were recruited from the same site, thus controlling for potential confounding effects of endemic disease. Furthermore, both HIV- and HIV+ subjects with acute illness or active malaria or tuberculosis infections, which can trigger perturbations in immune subsets, were excluded from the study.

While MAIT cells expand in the periphery after birth, it is unknown at what age MAIT cell frequencies reach adult levels. Lantz *et al*. suggested MAIT cell frequency steadily rises until age 2 years based upon observations in a limited number of infants, yet a larger cohort is necessary to better delineate the timeline [12, 14]. In one study on pediatric obesity, MAIT cells did not correlate with age in children between 5–18 years old. Similarly, in our cohort of HIV negative controls, there was no correlation between age and MAIT cell frequency in CD8+ T cells, suggesting that MAIT cells may reach adult levels during early childhood. However, the dynamics of the MAIT cell compartment and whether there are significant contractions or expansions throughout life remains unknown.

Our cohort of HIV-infected children from Kenya had clearly lower frequencies of CD8+ MAIT cells compared to their HIV negative healthy local age-matched controls. The consequences of such MAIT cell depletion in children may surmount those in adults, as immunologic immaturity renders even healthy children more susceptible to invasive bacterial infections, such as *Staphylococcus aureus* and mycobacterium. In resource-limited settings, despite antiretrovirals, HIV+ children have 30 times higher mortality than uninfected counterparts, mainly stemming from common childhood illnesses and opportunistic infections [49]. Studies from sub-Saharan African countries report the most common cause of death in HIV+ children on ART are bacterial pneumonia and diarrheal illnesses [49]. MAIT cells serve an integral role in clearance of such infections, and their depletion may contribute to related mortality in HIV+ children despite ART.

While multiple studies demonstrate decreased MAIT cells in HIV+ adults [15, 29–37], the mechanism of MAIT cell depletion remains unclear. In SIV infection, Vinton *et al*. demonstrated that MAIT cells are depleted from peripheral blood, with increased cell turnover but no evidence of increased migration to mucosal sites [37]. In perinatally-infected HIV+ children, one possible explanation for low circulating MAIT levels is that MAIT cells may have failed to expand normally in infancy and early childhood. Further studies of infants with perinatally-acquired HIV are necessary to evaluate this possibility. Interestingly, MAIT cells showed no association with CD4 percentages or HIV viral load. Other studies have demonstrated that CD8 MAIT cells are not infected by HIV *in vivo*, despite high frequencies of CCR5 expressed on their cell surface [15]. However, in HIV+ children, CD8 MAIT cells were specifically reduced in subjects with CD4:CD8 ratios less than one, which is a predictor of HIV mortality. Additionally declining CD8 MAIT cells correlated with increasing sCD14 that serves as a proxy for evidence of microbial translocation and also independently predicted HIV mortality in adults [44]. Taken together, these associations suggest a sharper decline of CD8 MAIT cells may link to HIV morbidity and mortality.

Several studies in adults report an irreversible decrease of MAIT cells in HIV+ subjects, even after antiretroviral therapy [15, 29, 31, 32]. Remarkably HIV+ children in this cohort had a small increase of CD8+ MAIT cell frequency after one year of treatment. Moreover, prolonged antiretroviral therapy over many years predicted higher frequencies of MAIT cells. The median increase in MAIT cells was 0.27 after the first year of treatment in ART- children, and 0.15 with continued treatment after one year in ART+ children. In our cross-sectional analysis, the difference between the median frequencies of CD8 MAIT cells in HIV+ and HIV negative children was 1.1. Thus, recovery of this subset may entail eight years of treatment if there is an increase of 0.15 each year. The low frequencies of MAIT cells that we observed in ART+ children may be due to inadequate duration of ART, as the median time on ART in this cohort was 2.6 years. We also determined that an additional factor in the recovery of MAIT cells is the age at which ART is initiated. Indeed, earlier ART initiation predicts a larger fold increase in CD8 MAIT cells. We speculate that with early ART initiation and prolonged treatment, HIV+ children may be able to recover CD8 MAIT cells. The recent recommendation from the World Health Organization recommending HIV treatment at the time of diagnosis may facilitate restoration of MAIT cells in children, and perhaps reduce bacterial infections and associated mortality in HIV+ children [50].

CD8 MAIT cell frequency was also closely correlated with peripheral neutrophils in HIV-infected children. Davey *et al*. recently demonstrated prolonged survival and differentiation of neutrophils into APC-like cells after stimulation when cultured with activated MAIT cells [51]. Accordingly, supernatants from neutrophils cultured with riboflavin producing bacteria activated a cytokine response in MAIT cells, thus demonstrating a reciprocal relationship between MAIT cells and neutrophils [51]. Indeed, the correlation we found between MAIT cells and neutrophils in HIV+ children, showed a similar trend in HIV negative children. In this study we also demonstrated severe disruption of other innate-like T cells, which confer protection from pathogenic bacteria. NKT cells, which share transcription factor PLZF with MAIT cells and also mediate antibacterial functions, were markedly reduced in both ART- and ART+ children. These findings differ from Chiappini’s report of stable CD4- NKT cells in HIV+ children on suppressive ART [52]. In our study, MAIT cells and NKT cell frequencies were both decreased and correlated in HIV negative children. Conversely, we found elevated DN γδ T cells in HIV+ children, similar to a previous report [53]. γδ T cells also produce cytokines IFNγ and TNFα in responses to bacterial infections. The rise in γδ T cells may reflect a compensatory response to decreased peripheral MAIT cells, supported by the inverse correlation between these populations. These multiple associations between CD8 MAIT and innate and innate-like cells suggest an intimate cross talk between these antibacterial effector populations during HIV infection.

Interestingly, MAIT cells share phenotypic features of Th17 cells, which are also CD161^+^CCR6^+^ and secrete IL-17. The essential antibacterial and antifungal functions of Th17 cells were shown in patients with autosomal dominant hyper-IgE syndrome characterized by STAT3 mutations, who had a complete loss of Th17 cells and presented with recurrent staphylococcal skin infections and mucocutaneous candidiasis [54]. Recently, STAT3 deficiency with loss-of-function mutations in IL12RB1 was shown to also result in decreased MAIT cells with impaired IL-17 production [55]. Thus, using STAT3 deficient patients as a model, the simultaneous loss of both Th17 and MAIT cells may explain increased susceptibility to mucocutaneous candidiasis and invasive infections due to *Staphylococcus aureus* during HIV infection. We found decreased Th17 cells in treated HIV+ children, but untreated HIV+ subjects maintained their Th17 cell subsets. Indeed, we previously described similar changes to Th17 cells in treated HIV-infected adults [56]. One possible explanation for preservation of Th17 cells in the ART- cohort may be a higher inflammatory state with a cytokine milieu promoting Th17 cell differentiation [57]. Additionally, while our ART- cohort had persistent HIV viremia and low CD4 percentages, we note this is a naturally selected cohort that survived in the absence of antiretroviral therapy into late childhood in a region where untreated HIV infection nears a fifty percent mortality rate by the age of 2 years [58]. Thus, the preservation of Th17 cells in this ART- cohort may reflect an immune protective mechanism that promotes this survival.

In the ART+ cohort, Th17 cells were depleted in both IFNγ^+^ and IFNγ^-^ IL-17A^+^ subsets. Interestingly, Zielinski *et al*. demonstrated *Candida albicans* specific Th17 cells produce IFNγ whereas *Staphylococcus aureus* specific Th17 cells produce IL-10 [59]. In our cohort, peripheral CD8 MAIT cell frequencies correlated with IFNγ^+^IL-17^+^ cells, matching the cytokine profile of *C*. *albicans* specific Th17 cells. Similar to Th17 subsets, Th22 cells are also essential to maintaining endothelial barriers by mediating host defense to enteropathogenic bacteria [48]. Moreover, IL-22 production by innate lymphoid cells is necessary to contain commensal bacteria within the gut lumen [60]. In our cohort, Th22 cells were depleted in HIV+ children and linked to a reduction in MAIT cells. Th1 cells were also decreased in treated HIV+ children, but had no association with MAIT cell frequencies. These correlations between MAIT cells with Th17 and Th22 populations but not with Th1 cells suggest a coordinated regulation of MAIT cells and other tissue-protective antibacterial effector T cell subsets concurrently disrupted by HIV infection. These simultaneous disruptions may account for increased mortality and susceptibility to bacterial infections in HIV+ children, especially in sub-Saharan Africa, despite adequate antiretroviral treatment.

In conclusion, we demonstrated a marked reduction of peripheral CD8 MAIT cell frequencies in perinatally-infected HIV+ children. These CD8 MAIT cells gradually increase with prolonged antiretroviral therapy, with greater recovery when ART is initiated at a young age. Diminished CD8 MAIT cells link to predictors of HIV mortality, low CD4:CD8 ratios and elevated plasma sCD14 levels. Moreover, CD8 MAIT cell frequencies closely correlate with innate and adaptive immune cells which mediate host defense against bacterial pathogens, including neutrophils, γδ, NKT, Th17, and Th22 T cells. Thus, HIV orchestrates several disruptions to innate defense and mucosally protective T cells with antibacterial effector functions. The multiple associations between MAIT cells and both innate and adaptive immune subsets suggest one disruption to the homeostatic state of sensing and responding to pathogenic and commensal bacteria may trigger concerted imbalances to host defense mechanisms during HIV infection.

## Supporting Information

S1 Fig HIV specific cells in Vα7.2 positive and negative CD8 T cells. Comparison of HIV specific CD8 T cells in Vα7.2- and Vα7.2+ populations. HIV specific CD8 T cells were identified by MHC dextramer SLYNVATYL (Immudex) staining. Shown are MHC dextramer SLYNVATL positive cells within Vα7.2- and Vα7.2+ CD8 T cells.
(TIF)

S2 Fig Total MAIT cells in HIV negative and positive children. Vα7.2^+^CD161^+^ populations (MAIT cells) in (A) CD3+ T cells and (B) total lymphocytes. (C) MAIT cell absolute numbers in lymphocytes calculated as %MAIT in lymphocytes multiplied by absolute lymphocyte count.
(TIF)

S3 Fig Immunologic and Virologic response to ART in HIV+ children. (A) %CD4 in ART- children before and 10–21 months after ART initiation. (B) HIV log copies/ml in ART- children before and 10–21 months after ART initiation. Statistical analysis was calculated with the paired Wilcoxon matched-pairs signed rank test.
(TIF)

S4 Fig FACS plots and gating strategy to identify NKT and γδT cells. (A) Within lymphocytes, we gated on CD3+ T cells (left plot) and then the Vα24Jα18^+^ population to identify NKT cells (right plot). (B) Total lymphocytes were gated on CD3+ T cells (left plot) then CD4-CD8- T cells (middle). Within CD4-CD8- T cells (DN), cells were gated on the γδTCR^+^ subset to identify DN γδT cells (right plot).
(TIF)

S5 Fig Th1 and MAIT cells in HIV+ and HIV- children. (A) Comparisons of IL-17A-IFNγ^+^ Th1 cells in HIV-, ART-, and ART+ children. (B) Correlation graphs between CD8+ MAIT cells and IL-17A-IFNγ^+^ Th1 cells in HIV+ (closed circles) and HIV- (open circles) children. All cytokine populations were gated within CD45RO+ memory CD4+ T cells.
(TIF)

S1 Table Demographic and Clinical Characteristics of Subjects. (PDF)
