# Supplementary figures and images for: HIV-Infected Children Have Lower Frequencies of CD8+ Mucosal-Associated Invariant T (MAIT) Cells that Correlate with Innate, Th17 and Th22 Cell Subsets
Source: PLoS One. 2016 Aug 25;11(8):e0161786. doi: 10.1371/journal.pone.0161786 (PMC4999196; doi:10.1371/journal.pone.0161786)

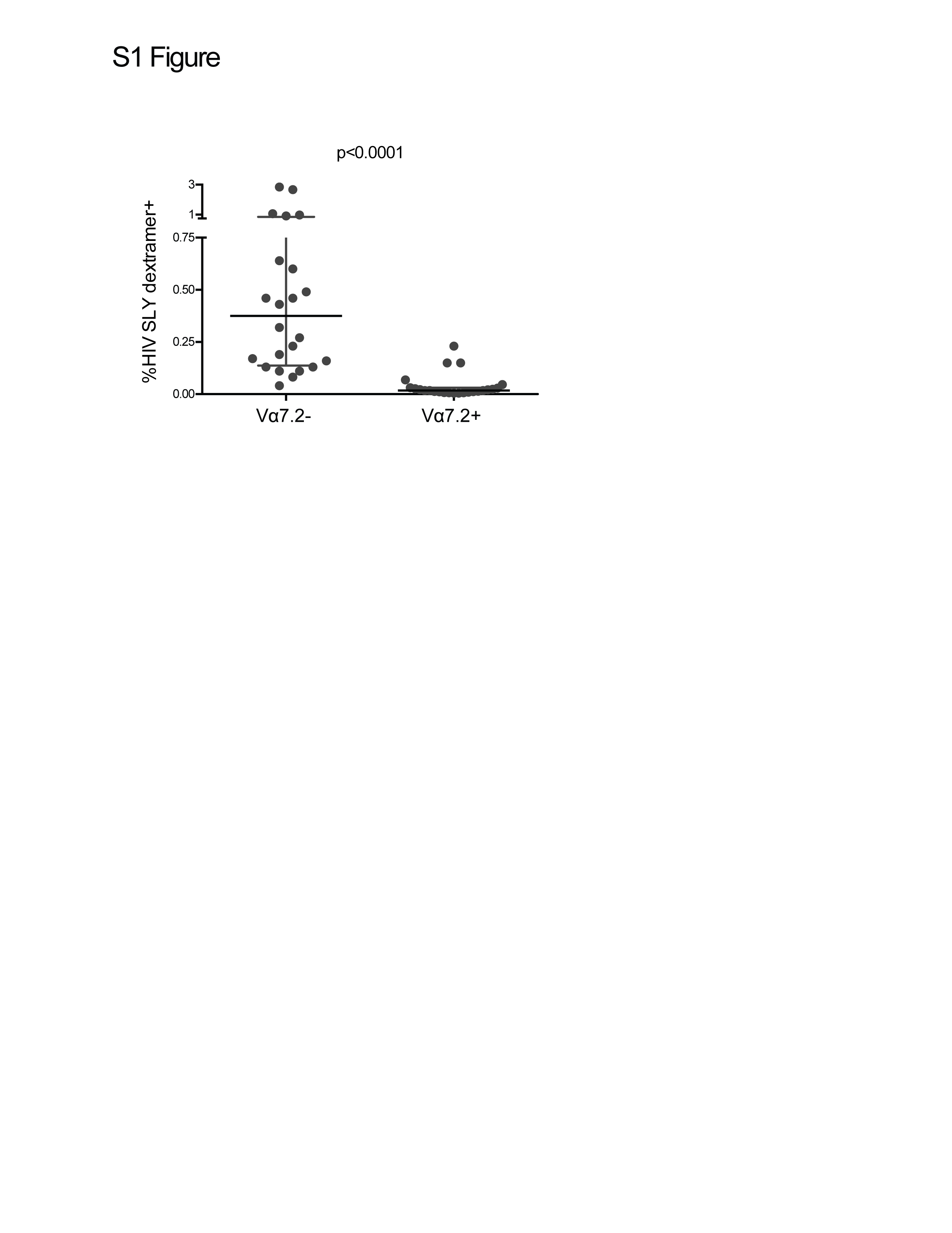

Supplement: S1 Fig — Comparison of HIV specific CD8 T cells in Vα7.2- and Vα7.2+ populations. HIV specific CD8 T cells were identified by MHC dextramer SLYNVATYL (Immudex) staining. Shown are MHC dextramer SLYNVATL positive cells within Vα7.2- and Vα7.2+ CD8 T cells. (TIF) [file pone.0161786.s001.tif]

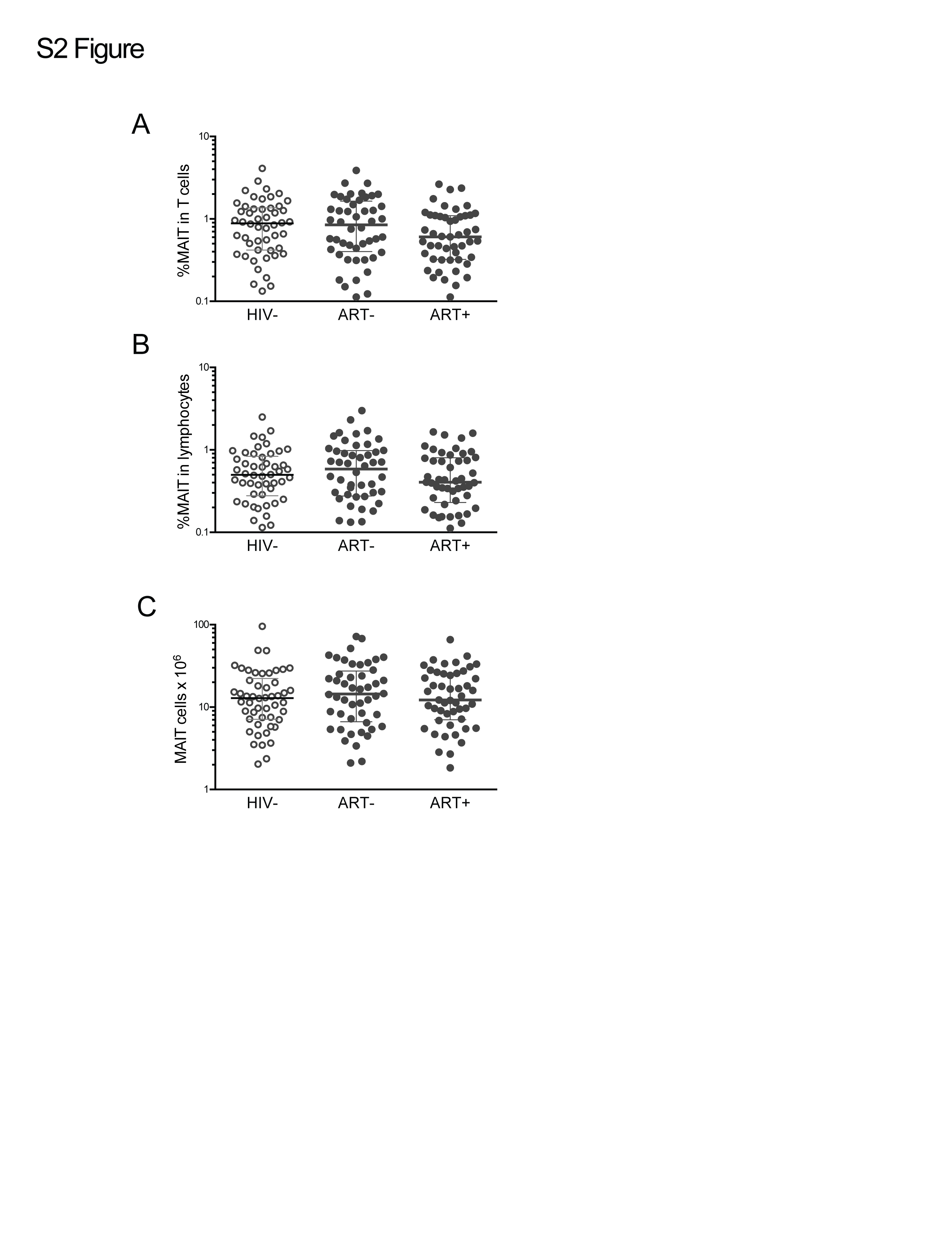

Supplement: S2 Fig — Vα7.2+CD161+ populations (MAIT cells) in (A) CD3+ T cells and (B) total lymphocytes. (C) MAIT cell absolute numbers in lymphocytes calculated as %MAIT in lymphocytes multiplied by absolute lymphocyte count. (TIF) [file pone.0161786.s002.tif]

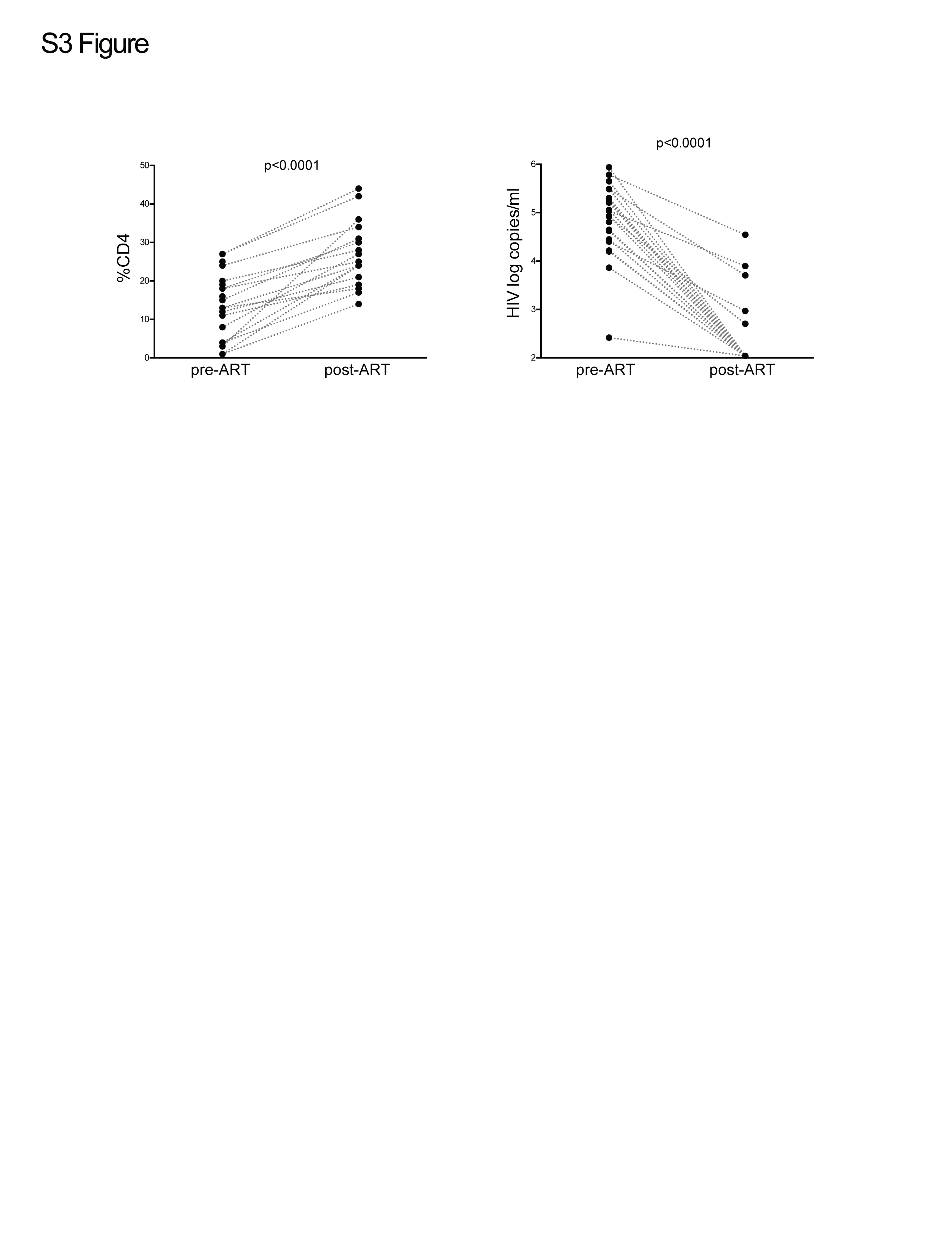

Supplement: S3 Fig — (A) %CD4 in ART- children before and 10–21 months after ART initiation. (B) HIV log copies/ml in ART- children before and 10–21 months after ART initiation. Statistical analysis was calculated with the paired Wilcoxon matched-pairs signed rank test. (TIF) [file pone.0161786.s003.tif]

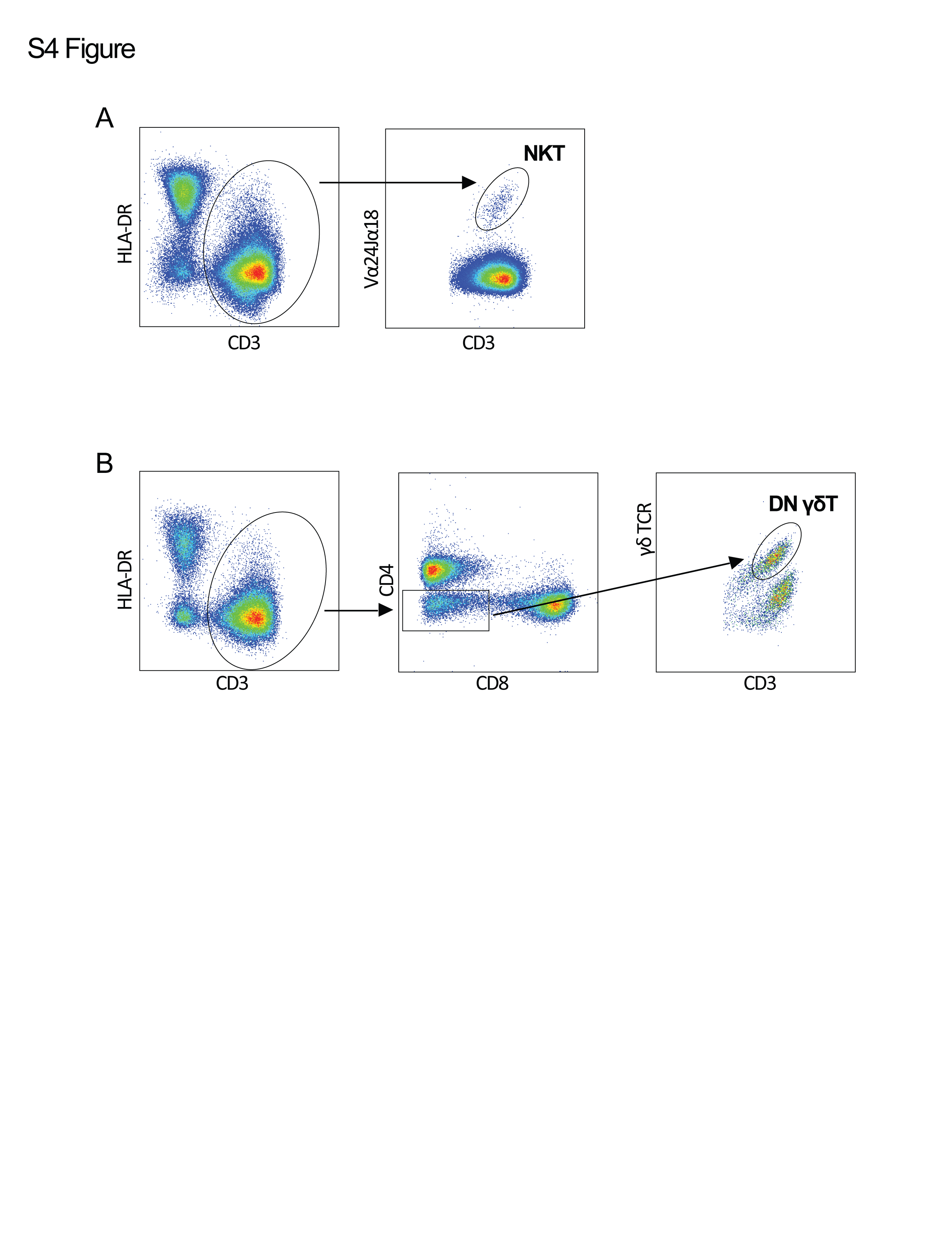

Supplement: S4 Fig — (A) Within lymphocytes, we gated on CD3+ T cells (left plot) and then the Vα24Jα18+ population to identify NKT cells (right plot). (B) Total lymphocytes were gated on CD3+ T cells (left plot) then CD4-CD8- T cells (middle). Within CD4-CD8- T cells (DN), cells were gated on the γδTCR+ subset to identify DN γδT cells (right plot). (TIF) [file pone.0161786.s004.tif]

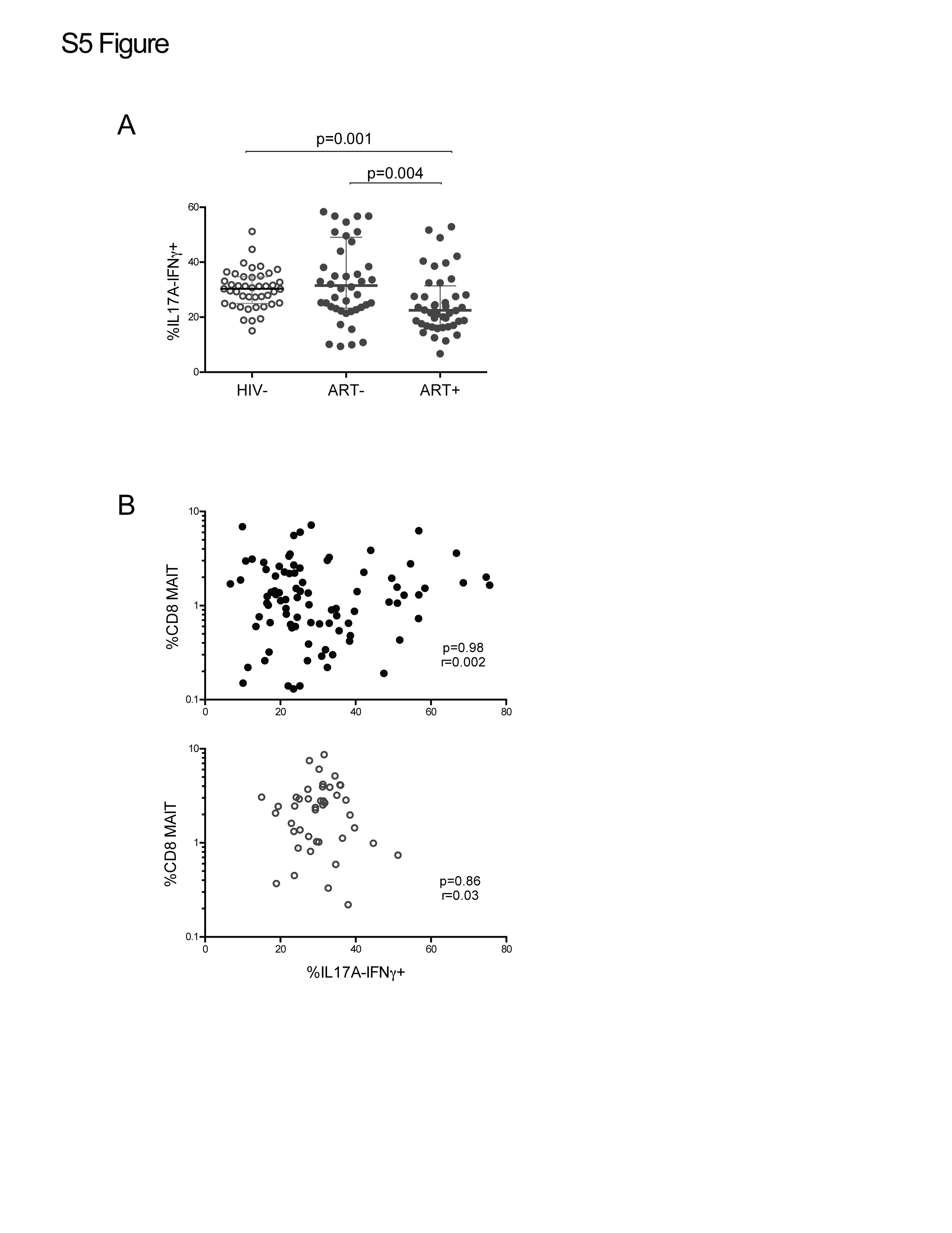

Supplement: S5 Fig — (A) Comparisons of IL-17A-IFNγ+ Th1 cells in HIV-, ART-, and ART+ children. (B) Correlation graphs between CD8+ MAIT cells and IL-17A-IFNγ+ Th1 cells in HIV+ (closed circles) and HIV- (open circles) children. All cytokine populations were gated within CD45RO+ memory CD4+ T cells. (TIF) [file pone.0161786.s005.tif]
