# Supplementary material for: HIV-Infected Children Have Lower Frequencies of CD8+ Mucosal-Associated Invariant T (MAIT) Cells that Correlate with Innate, Th17 and Th22 Cell Subsets
Source: PLoS One. 2016 Aug 25;11(8):e0161786. doi: 10.1371/journal.pone.0161786 (PMC4999196; doi:10.1371/journal.pone.0161786)
Supplement: S1 Table — (PDF) [file pone.0161786.s006.pdf]

S1 Table: Subjects' Demographic and Clinical Characteristics

PID	Category	Sex	Age (years)	%CD4	CD4 cells/mm3	HIV copies/mL	HIV log copies/mL
18	HIV-	M	8.6	38	741		
36	HIV-	M	6.4	33	1226		
39	HIV-	M	8.2	33	546		
40	HIV-	F	4.8	39	571		
58	HIV-	M	18.2	30	578		
62	HIV-	M	15.6	28	548		
63	HIV-	M	17.6	30	581		
65	HIV-	M	17.4	33	1522		
66	HIV-	M	17.1	29	830		
68	HIV-	F	3.8	33	1444		
69	HIV-	F	3.5	40	1899		
70	HIV-	F	11.7	46	908		
71	HIV-	F	3.4	31	1376		
75	HIV-	F	15.7	39	1042		
82	HIV-	M	4.0	27	902		
83	HIV-	M	5.5	36	767		
84	HIV-	F	10.7	42	937		
90	HIV-	F	15.8	37	982		
91	HIV-	M	11.9	36	1146		
92	HIV-	M	9.5	25	408		
93	HIV-	F	4.0	26	1091		
99	HIV-	F	4.0	27	1143		
103	HIV-	M	10.9	39	1228		
104	HIV-	M	3.6	38	1220		
106	HIV-	M	6.7	43	897		
107	HIV-	M	11.7	37	1830		
108	HIV-	F	10.8	38	965		
109	HIV-	F	8.1	48	1461		
111	HIV-	M	11.1	43	1382		
112	HIV-	F	6.6	51	1896		
115	HIV-	F	7.1	43	1305		
116	HIV-	F	12.6	45	1099		
117	HIV-	F	9.3	38	1133		
118	HIV-	M	3.8	22	535		
119	HIV-	F	3.8	43	1135		
122	HIV-	M	17.6	38	1383		
123	HIV-	F	13.3	29	1681		
124	HIV-	M	11.1	45	2213		
125	HIV-	M	15.7	14	604		
128	HIV-	M	12.3	33	611		
129	HIV-	M	11.0	34	601		
143	HIV-	M	14.0	40	827		
144	HIV-	F	17.7	40	674		
149	HIV-	F	11.1	42	1093		
152	HIV-	F	10.7	29	746		
159	HIV-	M	6.8	25	818		
161	HIV-	M	12.5	34	880		
162	HIV-	M	3.1	30	1325		
230	HIV-	M	10.5	44	732		
231	HIV-	F	11.8	40	957		
234	HIV-	M	11.4	37	576		
237	HIV-	F	3.5	22	1303		
14	ART-	F	4.0	29	1347	34,155	4.5
15	ART-	M	9.9	1	17	263	2.4
28	ART-	F	9.3	18	583	533,500	5.7
31	ART-	M	9.1	26	1240	279,400	5.4
37	ART-	F	3.6	36	1764	1,947	3.3

PID	Category	Sex	Age (years)	%CD4	CD4 cells/mm3	HIV copies/mL	HIV log copies/mL
38	ART-	M	10.6	5	340	163,350	5.2
44	ART-	M	15.8	35	993	3,586	3.6
48	ART-	F	6.7	42	1294	65,450	4.8
59	ART-	M	10.6	30	902	83,050	4.9
72	ART-	M	8.7	8	252	176,550	5.2
77	ART-	F	6.6	37	1309	187,000	5.3
87	ART-	F	14.8	18	531	144,650	5.2
88	ART-	F	7.7	27	625	306,350	5.5
94	ART-	M	7.4	20	991	27,940	4.4
95	ART-	M	13.7	37	738	19,855	4.3
101	ART-	F	9.2	34	767	4,675	3.7
113	ART-	M	7.3	24	669	20,185	4.3
114	ART-	F	8.8	27	1265	36,465	4.6
134	ART-	M	7.8	18	858	304,150	5.5
135	ART-	F	8.8	25	812	168,850	5.2
141	ART-	M	5.6	15	591	7,315	3.9
142	ART-	F	11.8	28	497	6,710	3.8
145	ART-	M	6.0	13	524	113,300	5.1
146	ART-	F	9.0	18	648	42,130	4.6
147	ART-	F	11.0	26	494	30,910	4.5
157	ART-	M	9.1	12	286	442,750	5.6
167	ART-	F	8.1	16	545	160,875	5.2
169	ART-	M	8.5	24	383	199,375	5.3
174	ART-	F	12.4	22	514	2,728	3.4
178	ART-	M	6.9	28	1067	122,100	5.1
180	ART-	F	14.9	27	505	80,850	4.9
184	ART-	M	11.6	13	285	16,500	4.2
198	ART-	M	3.4	11	478	605,000	5.8
199	ART-	F	14.2	25	502	64,570	4.8
202	ART-	F	12.7	27	396	15,730	4.2
203	ART-	F	12.0	1	18	83,050	4.9
204	ART-	F	11.5	33	630	7,989	3.9
205	ART-	F	14.0	4	71	109,450	5.0
208	ART-	F	3.9	21	1127	5,995	3.8
209	ART-	F	13.7	19	352	25,245	4.4
210	ART-	M	3.9	9	257	246,400	5.4
212	ART-	F	3.1	33	1543	227,700	5.4
214	ART-	M	14.9	4	173	863,500	5.9
217	ART-	M	16.3	5	44	303,600	5.5
219	ART-	F	10.7	43	755	14,200	4.2
220	ART-	F	13.0	29	705	40,425	4.6
221	ART-	M	17.1	28	645	84,700	4.9
224	ART-	M	14.6	3	175	44,165	4.6
225	ART-	M	3.4	12	437	85,250	4.9
3	ART+	F	9.5	36	861	459	2.7
4	ART+	M	12.5	28	970	110	2.0
7	ART+	F	6.2	25	1497	2,772	3.4
8	ART+	F	13.0	30	765	110	2.0
9	ART+	M	9.8	51	816	110	2.0
13	ART+	M	12.9	47	1221	110	2.0
22	ART+	M	12.7	18	616	110	2.0
23	ART+	F	7.5	32	919	110	2.0
26	ART+	F	3.7	34	1271	110	2.0
27	ART+	F	5.3	43	2634	110	2.0
29	ART+	F	18.5	26	492	100	2.0
30	ART+	F	9.7	41	700	110	2.0
32	ART+	M	3.7	32	980	110	2.0

PID	Category	Sex	Age (years)	%CD4	CD4 cells/mm3	HIV copies/mL	HIV log copies/mL
33	ART+	F	9.1	23	927	110	2.0
34	ART+	M	7.5	32	784	110	2.0
35	ART+	F	6.6	40	2062	110	2.0
45	ART+	M	4.0	26	1790	1,914	3.3
46	ART+	M	10.6	41	1234	110	2.0
47	ART+	M	12.5	13	153	9,460	4.0
49	ART+	M	9.0	35	931	110	2.0
50	ART+	M	8.4	43	735	110	2.0
51	ART+	F	5.7	30	962	110	2.0
52	ART+	F	13.1	26	651	110	2.0
53	ART+	F	15.6	32	560	110	2.0
54	ART+	M	4.6	44	1703	110	2.0
55	ART+	M	3.6	30	1794	110	2.0
56	ART+	F	8.2	41	1818	110	2.0
60	ART+	M	11.8	10	109	110	2.0
61	ART+	M	6.8	34	1037	110	2.0
73	ART+	F	11.7	32	1412	218	2.3
74	ART+	F	13.1	24	972	743	2.9
76	ART+	M	9.2	30	1202	110	2.0
86	ART+	F	17.8	20	522	1,711	3.2
89	ART+	M	3.5	26	1014	3,372	3.5
96	ART+	M	17.0	14	201	6,105	3.8
105	ART+	M	13.1	7	374	26,400	4.4
120	ART+	M	13.9	38	1572	110	2.0
132	ART+	F	13.6	33	1116	110	2.0
136	ART+	F	11.8	43	1338	130	2.1
138	ART+	M	14.9	30	1095	110	2.0
139	ART+	F	12.5	33	567	2,877	3.5
140	ART+	M	16.7	20	477	13,090	4.1
153	ART+	F	3.3	27	1658	110	2.0
154	ART+	M	3.5	28	1297	110	2.0
156	ART+	M	3.8	29	1361	110	2.0
164	ART+	F	3.1	47	2751	110	2.0
170	ART+	F	3.3	35	1763	110	2.0
232	ART+	F	3.4	34	1068	110	2.0
238	ART+	M	3.5	31	1072	2,552	3.4
